# Ten years’ experience with bone conduction hearing aids in the Western Cape, South Africa

**DOI:** 10.4102/sajcd.v70i1.940

**Published:** 2023-01-27

**Authors:** Silva Kuschke, Christine Rogers, Estie Meyer

**Affiliations:** 1 Department of Audiology, Faculty of Rehabilitation Sciences, Red Cross War Memorial Children’s Hospital Cape Town, Western Cape, South Africa; 2 Department of Health and Rehabilitation Sciences Faculty of Communication Sciences and Disorders, University of Cape Town, Cape Town, South Africa; 3 Department of Otorhinolaryngology, Faculty of Health Sciences, University of Cape Town, Cape Town, South Africa

**Keywords:** bone conduction hearing aids, hearing loss, softband, abutment, accessible hearing healthcare

## Abstract

Untreated conductive and mixed hearing losses as a result of middle ear pathology or congenital ear malformations can lead to poor speech, language and academic outcomes in children. Lack of access to centralised hearing healthcare in resource-constrained environments limits opportunities for children with hearing loss. Red Cross War Memorial Children’s Hospital (RCWMCH) is one of only two dedicated paediatric hospitals in sub-Saharan Africa. Between 2016 and 2021, 29 children received implanted bone conduction hearing devices, and 104 children were fitted with bone conduction devices on softbands. The authors’ experience at RCWMCH suggests that bone-anchored hearing devices, either fitted on softbands or on implanted abutments, can provide solutions in settings where patients have limited access to hearing healthcare and optimal classroom environments. Hearing healthcare should be accessible and delivered at the appropriate level of care to mitigate the adverse effects of hearing loss in children.

**Contribution:**

This article describes strategies employed at RCWMCH such as fitting bone conduction hearing devices on a softband immediately after hearing loss diagnosis and conducting follow-up via remote technology to make hearing healthcare more accessible to vulnerable populations.

## Introduction

### Preventable childhood hearing loss in low- to middle-income countries

The World Health Organization (WHO, 2021) estimates that more than 60% of hearing loss in children under the age of 15 years is preventable, with this figure being substantially higher in low- to middle-income countries (LMICs). The dramatic increase in preventable hearing loss in under-resourced countries may be attributed to limited access to resources such as vaccines, as well as a possible predisposition to infections such as otitis media and associated hearing loss (Olusanya et al., 2019; WHO, 2021). The most common causes of childhood conductive hearing loss (CHL) are related to infection or structural abnormalities of the outer and/or middle ear (Kesser et al., 2013). Otitis media occurs in approximately 50% of children under the age of one year and 60% of children under the age of two years (Simon et al., 2018). Although episodic acute cases of otitis media are common during childhood, recurrent otitis media can affect hearing throughout childhood, with the associated degree of CHL generally correlating with the severity of the infection (Jensen et al., 2013). The resultant hearing loss is often caused by persistent fluid in the middle ear cavity, tympanic membrane perforations or corrosion of the ossicular chain (Hunter & Choo, 2017). Single-sided hearing loss is defined as unilateral hearing loss which is characterised by a severe-to-profound hearing loss in one ear (pure-tone average threshold ≥ 65 dBHL) and normal hearing (pure-tone average threshold < 30 dBHL) in the contralateral ear (WHO, 2021). Children with single-sided hearing loss have difficulty with sound localisation, as well as understanding speech, especially with background noise. There are several causes for single-sided hearing loss in children, including idiopathic sudden sensorineural hearing loss, congenital cytomegalovirus, meningitis and head trauma (Usami et al., 2017).

### Management of childhood hearing loss in low- to middle-income countries

The adverse effects of hearing loss, irrespective of its aetiology, on childhood development manifest as delays in speech and language development (Gouma et al., 2011). Furthermore, behavioural problems (such as attention deficits, failure to engage and respond during conversational discourse and a lack of interest in communication) could lead to social maladaptation and poor academic performance (Gouma et al., 2011). Internalised behavioural problems such as anxiety and depression have been reported in children with CHL (Bush, 2019). Thus, the desire to manage hearing loss as early as practicable and as holistically as possible is essential, particularly in paediatric populations. Certain syndromes, which are more likely to occur in LMICs, may predispose children to conductive or mixed hearing loss (Kesser et al., 2013). These syndromes include Down syndrome, Crouzon syndrome and Goldenhar syndrome. Congenital structural conditions of the pinna and outer ear such as atresia and microtia might prevent prescription of conventional behind-the-ear hearing aids. In addition, children with chronic otorrhoea are not candidates for air conduction hearing aids, and bone conduction devices may be a more suitable option.

### Bone conduction hearing devices

Implantable bone conduction hearing devices were pioneered in the seventies (Faber et al., 2015). Bone conduction hearing aids are amplification devices that conduct sound to the cochlea through the transmission of vibrations from a sound processor to the mastoid (Westerkull, 2018). The device bypasses the middle ear and is traditionally used in patients with CHL who are unable to use air conduction hearing aids (Liu et al., 2017). Bone conduction hearing aids can transmit sound transcutaneously if the sound processor is anchored to the head by an external fabric headband (commonly referred to as a softband) or percutaneously if the sound processor is attached to a titanium fixture which is surgically implanted in the skull (Mejia et al., 2015). In developed countries, bone conduction devices can be implanted in two stages in patients as young as four years (Davids et al., 2007; Humitz, 2016; Westerkull, 2018). In LMICs such as South Africa, operating time is often limited, and theatre waiting lists are long, especially following the devastating impact of the coronavirus disease 2019 (COVID-19) pandemic (Klazura et al., 2022). Therefore, permanent bone conduction implants are performed at eight years for typically developing children and at 10 years for syndromic children with thinner skull measurements. In the meantime, children continue to wear their bone conduction device on a softband to have access to sound.

**FIGURE 1:**
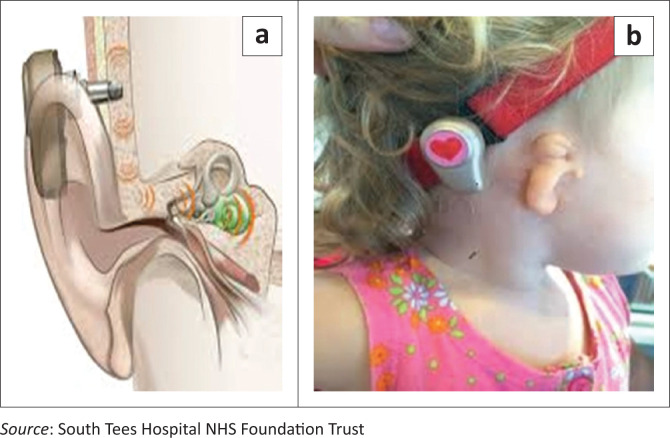
Percutaneous bone conduction device (a) and transcutaneous bone conduction device on a softband (b).

Figure 1 shows a schematic representation of the abutment and device on the left and a softband worn by a child with a malformed pinna on the right.

Current paediatric guidelines in the Western Cape province for the management of persistent conductive, mixed or single-sided hearing losses include medical or surgical treatment, as well as the use of bone conduction hearing devices.

The next section of this article presents data from Red Cross War Memorial Children’s Hospital (RCWMCH) in the Western Cape province of South Africa over six years, followed by recommendations for accessible service delivery in resource-constrained settings.

## Method

Red Cross War Memorial Children’s Hospital is one of only two dedicated paediatric tertiary-level academic hospital in sub-Saharan Africa and serves as a central referral hospital for patients across the entire Western Cape who require specialised healthcare services. The audiology department at RCWMCH provides diagnostic audiology and intervention services to children from birth to 13 years from the public healthcare sector.

### Study design and sampling

A retrospective review of clinical data, using an electronic departmental database, was conducted. Purposive sampling was used by the primary investigator to identify children between 0 and 13 years, who were fitted with bone conduction hearing devices, either on a softband or implantable abutment, between January 2016 and December 2021. The departmental database was established in 2016; therefore, data were reviewed from 2016 to 2021 to include a wide lens of records.

### Data collection and analysis

Participants were identified retrospectively using an electronic departmental database from which their demographic and clinical information was recorded. Data were anonymised by assigning an alphanumeric code to each participant.

All data were captured on an Excel spreadsheet, using Microsoft Excel 2022 (Microsoft Corporation, Redmond, Washington, United States). The data were analysed using SPSS 27, version 27.0 (IBM Corporation, Armonk, New York, United States). Descriptive analysis was used to report on the findings.

### Ethical considerations

This article followed all ethical standards for research without direct contact with human or animal subjects.

## Results

The retrospective departmental database review indicated that a total of 9215 children were seen by the audiology department at RCWMCH during January 2016 and December 2021. Of these, 498 children were fitted with amplification devices, of which 133 were bone conduction hearing devices on softbands (*n* = 104) and permanent implanted abutments (*n* = 29). Table 1 details recipients of bone conduction devices.

**TABLE 1:** Profile of patients fitted with implanted and softband bone conduction devices at Red Cross War Memorial Children’s Hospital from 2016 to 2021.

Devices issued per type of HL	2016	2017	2018	2019	2020	2021	Total
**Permanent bone conduction implants**							
Total devices	6	4	5	4	3	7	29
Conductive HL	3	3	1	1	1	3	12
Single-sided HL	1	1	3	3	0	2	10
Mixed HL	2	0	1	0	2	2	7
Mean age in years (range)	8 (7–10)	8 (8–11)	9 (8–12)	9 (9–12)	11 (11–13)	9 (8–12)	-
**Bone conduction devices on softbands**							
Total devices	13	22	11	24	14	20	104
Conductive HL	7	10	5	13	12	12	59
Single-sided HL	4	7	5	6	1	3	26
Mixed HL	2	5	1	5	1	5	19
Mean age in years (range)	4 (1–6)	5 (2–10)	5 (2–7)	4 (0.5–12)	5 (2–12)	6 (3–13)	-

*Source:* RCWMCH Audiology Department

HL, hearing loss.

The majority of patients who received either implanted or softband bone conduction hearing devices during 2016 – 2021 presented with CHL (53.4%; *n* = 71). Nearly one-third of our sample (27.1%; *n* = 36) presented with single-sided hearing loss. It is notable that during 2020, when the COVID-19 pandemic was at a peak globally, the number of bone conduction implants and devices issued on softbands decreased. The decrease was likely because of audiology outpatient services de-escalating and patients having restricted access to nonemergency healthcare services.

## Discussion

Success in many areas of healthcare in LMICs speaks directly to how accessible and patient-centred services are. For example, patients in LMICs often travel great distances to access centralised hearing services (Olusanya, 2015), and upon receiving them, they might struggle to return for regular follow-up. The audiology service at RCWMCH has addressed these challenges in the following ways:

- Patients are fitted with bone conduction devices on softbands prior to abutment implantation, and device usage (measured in average hours per day) is documented via data logging software in the device. Patients who present with CHL caused by tympanic membrane perforations or chronically leaking ears, and who are too young for surgical intervention, are fitted with bone conduction devices on a softband until they are old enough for surgery. The bone conduction processor fitted to a softband means that the device can be issued on the day of diagnosis. This rapid response speaks to the principles of primary healthcare in terms of accessibility and acceptability.
- Functional hearing outcomes are measured by validated parent–teacher feedback questionnaires, such as the Parents’ Evaluation of Aural/Oral Performance of Children (PEACH) and the Teachers’ Evaluation of Aural/Oral Performance of Children (TEACH) (Ching & Hill, 2005; National Deaf Children’s Society, [no date]). The PEACH and TEACH are measures of everyday functional auditory and communication performance and can be used to identify situations that could negatively impact a child’s regular amplification use (Ching & Hill, 2005). Both questionnaires require the caregiver or teacher to observe and rate both the child’s listening and communication skills in quiet and noisy real-life situations and were designed for use for children of all ages (Ching & Hill, 2005).
- Affordability is crucial in primary healthcare. In this instance, funding for 20 bone conduction hearing devices per annum was secured by the Western Cape Provincial Department of Health in 2014, so that no end-user point of payment is necessary. The authors acknowledge that access to funding has greatly accelerated programme rollout and are grateful that the political will to provide such care is present in the region.
- Wound care follow-up for patients who received permanent abutment implants is optionally facilitated by the use of technology. WhatsApp pictures of abutment complications (such as skin infection around the implanted site) can be sent to the ear, nose and throat (ENT) surgeon, who can refer the patient directly to the appropriate level of care (primary-level general practitioner). Patients’ travelling distance to a centralised tertiary service can be reduced in this way, and infections that arise as a complication of the abutment surgery can be treated timeously. For patients who do not have access to a smartphone or mobile data, written information on wound care is given, and they are advised to come to the hospital immediately if any postoperative wound complications (such as skin redness or crusting) are observed.
- South African classrooms are often large and noisy, and resources such as FM systems and remedial support are not routinely available in many public schools. In order to facilitate inclusivity within the South African public education sector, bone conduction devices are offered to children with single-sided hearing loss, where the bone conduction device acts as a cross-router for better localisation. Bone conduction devices are often issued for children with single-sided hearing loss based on teacher feedback of poor scholastic performance. Functional listening progress (especially in noise) is measured by the TEACH questionnaire, as described earlier. Such a strategy manages the often-overlooked issue of unilateral hearing loss, which has a negative impact on children’s social and academic advancement.

## Recommendations

The above description of a system to manage hearing healthcare in a resource-constrained environment has implications for planning and policy in similar settings in South Africa and other sub-Saharan African facilities. A review of the guidelines for medical and audiological treatment of conductive, mixed or single-sided hearing loss in children is necessary, especially when delayed speech development and poor scholastic performance are reported.
